# Psychosocial and Sociodemographic Factors Associated with Wrist Pain Severity and Dysfunction in Turkish Housewives: A Web-Based Cross-Sectional Survey

**DOI:** 10.3390/healthcare14091162

**Published:** 2026-04-26

**Authors:** Özlem Akkoyun Sert, Ece Ekici, Ümit Yüzbaşıoğlu

**Affiliations:** 1 Department of Physical Therapy and Rehabilitation, Faculty of Health Sciences, KTO Karatay University, 42000 Konya, Turkey; ozlem.sert@karatay.edu.tr; 2 Department of Physical Therapy and Rehabilitation, Faculty of Health Sciences, Toros University, 33140 Mersin, Turkey; 3 Department of Therapy and Rehabilitation, Vocational School of Health Services, Toros University, 33140 Mersin, Turkey; umit.yuzbasioglu@toros.edu.tr

**Keywords:** repetitive strain injury, housework, repetitive stress, fatigue, musculoskeletal disorders, household activity

## Abstract

**Background:** Wrist pain is frequently reported among housewives and linked to repetitive household tasks, yet the drivers of pain-related disability remain unclear. Beyond physical load, psychosocial factors such as catastrophizing, mood symptoms, and self-efficacy may shape severity and functional impact. **Purpose**: To evaluate the severity of wrist pain and wrist-related disability in Turkish housewives and to identify the psychosocial and symptom-related factors associated with these outcomes. **Methods:** This cross-sectional study was conducted with 92 Turkish housewives reporting wrist pain ≥ 3/10 on the Numeric Pain Rating Scale (NPRS) and who have been married for at least 1 year and performing at least 1 h of housework, via Google Forms. Fatigue and wrist pain were measured using the Numeric Rating Scale (NRS), as well as the Patient-Rated Wrist Evaluation–Turkish version (PRWE-T), Pain Self-Efficacy Questionnaire (PSEQ), Patient Health Questionnaire-4 (PHQ-4), and Pain Catastrophizing Scale (PCS). Sociodemographic data were also collected. Associations were analyzed with Spearman’s correlation, and simple linear regression identified factors explaining wrist pain severity and disability. **Results**: PRWE-T total scores showed strong positive correlations with pain catastrophizing (*p* < 0.001), PHQ-4 depression (*p* < 0.001), and PHQ-4 anxiety (*p* < 0.001), while correlating negatively with pain self-efficacy (*p* < 0.05). PCS was also strongly correlated with PHQ-4 anxiety (*p* < 0.001) and PHQ-4 total (*p* < 0.001), but negatively with PSEQ (*p* < 0.001). Multivariable regression analyses have shown that PCS and fatigue may be predictory of wrist pain and disability. Additional factors included fatigue severity (*p* = 0.002), PHQ-4 depression (*p* < 0.001), and PHQ-4 anxiety (*p* = 0.001). **Conclusions**: These findings highlight the multidimensional nature of wrist symptoms in this population.

## 1. Introduction

The processes contributing to the development of musculoskeletal complaints are influenced by various factors [1]. Research shows women have a higher incidence of repetitive stress injuries, especially in the upper extremities, compared to men [2,3,4]. Although the primary cause of this disparity is often attributed to anthropometric and physiological differences between the sexes, housework tasks usually contribute to this injury risk [5].

Women manage multiple roles within the home, performing diverse tasks involving various responsibilities that demand not only physical but also emotional and mental effort [6,7]. While housework is culturally esteemed in many societies, including Turkey, it carries undeniable health risks [8,9,10]. Housewives often engage in repetitive stress-inducing activities involving the upper extremities, particularly the wrists, such as washing dishes, cleaning windows, sweeping, mopping, and wringing out cloths. Ward’s early ergonomic analysis demonstrated that these domestic tasks frequently require prolonged static postures and repetitive upper-limb movements, leading to increased muscular strain during housework [11]. Both the intensity of these physical tasks and non-ergonomic conditions may contribute to the development of wrist pain and related disorders [12,13]. For example, the study by Mattioli et al. (2009) reported that housewives, similar to blue-collar workers, are at high risk for carpal tunnel syndrome [14]. The physical strain and biomechanical stress caused by these types of repetitive tasks can increase psychological and emotional burden, suggesting a potential interaction between musculoskeletal strain and mental health. Another study, by Habib et al. (2012), demonstrated a significant association between psychosocial factors and musculoskeletal pain in Lebanese housewives [15]. However, as Nawrin et al. (2020) reported, full-time homemakers seek medical attention related to musculoskeletal symptoms 40% less frequently than working women [16]. This contrast in health-seeking behavior may worsen symptoms and hinder the prognosis of musculoskeletal disorders, as housewives delay treatment, leading to prolonged pain and disability, which are often linked to negative psychological outcomes and further impact well-being [17].

Prior studies have focused on the prevalence of musculoskeletal disorders among housewives without focusing specifically on the wrists [9,18]. However, given that a significant proportion of housework involves repetitive stress concentrated on the wrists, it can be argued that wrist disorders in housewives may require a separate and detailed investigation. Existing evidence indicates wrist disorders are particularly common among housewives, with prevalence rates as high as 40.5% in some populations [19]. A further study, conducted by Yang et al. (2016), demonstrated the effects of housework on the hands and wrists [2]. The study revealed that approximately half of the women presenting to the clinic with repetitive stress injuries of the upper extremities were not working and argued that housework should be recognized as a form of employment.

Based on these studies, housekeeping may be considered a risk factor for wrist disorders; however, the factors influencing the frequently observed wrist complaints among housewives remain unknown. Therefore, the present study aimed to assess wrist pain severity and wrist-related disability and to investigate their psychosocial and symptom-related correlates in an online sample of Turkish housewives. Addressing this research gap is essential to better understand the multifaceted contributors to wrist pain in housewives and guide the development of proactive educational strategies, fostering early recognition of symptoms and encouraging timely interventions for housewives.

## 2. Methods

### 2.1. Study Design

**Figure 1:**
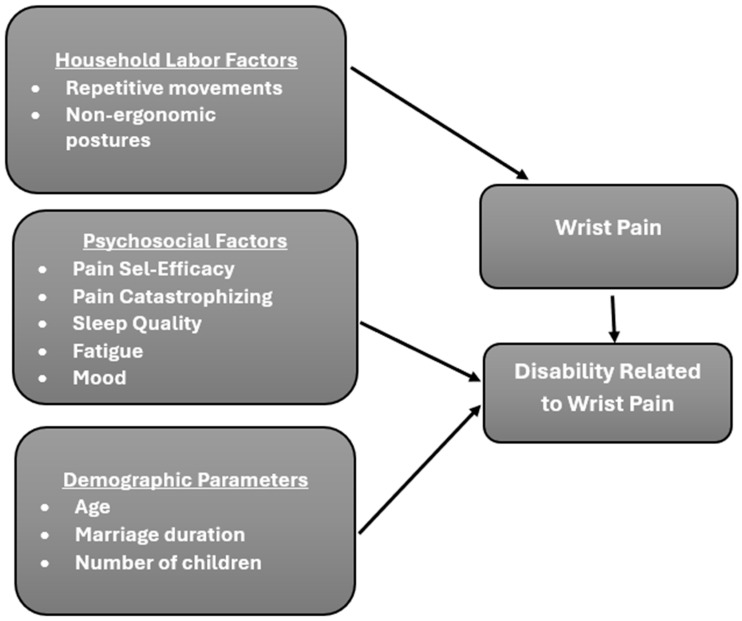
Conceptual framework.

This internet-based cross-sectional study followed the principles of the Checklist for Reporting Results of Internet E-Surveys (CHERRIES) [20] and the Strengthening the Reporting of Observational Studies in Epidemiology (STROBE) guidelines for observational studies [21]. Both reporting checklists are presented in Supplementary Materials (Supplementary Files S1 and S2). Prior to the study, ethical approval was obtained from the Toros University Faculty of Health Sciences Non-invasive Clinical Research Ethics Committee with the decision number 2024/9/27, dated 8 November 2024, and all procedures were carried out following the Declaration of Helsinki. A conceptual framework was developed to illustrate the hypothesized relationships among biomechanical, psychosocial, and demographic factors influencing wrist pain and related disability. This framework was based on prior literature indicating domestic workload and psychological stress could interact in the development of musculoskeletal disorders, and guided the selection of study variables and the analytical approach (Figure 1) [7]. G Power software (Heinrich-Heine-Universität Düsseldorf, Düsseldorf, Germany) Version 3.1.9.7 was used to calculate the sample size. Due to the absence of prior regression-based effect size estimates for hand–wrist pain among housewives, the sample size estimation was based on prevalence data to ensure an adequate number of cases for subsequent multivariable analysis. As a result of the effect size calculation, based on the prevalence of hand–wrist pain as 15% globally [22] and 32.3% in Turkey [23], it was determined that a minimum of 79 participants were needed with an effect size of 0.17, a 5% margin of error, and 95% power.

### 2.2. Participants

The data collection process was carried out online via Google Forms (https://forms.gle/13Wo4fgpaG7qJc198) URL (accessed on 20 February 2026) between 25 November 2024 and 25 December 2024 through a form consisting of 5 main sections in the form of an open and voluntary questionnaire using a convenience sampling method. This study was publicized as non-profit academic research, and the survey link was shared via social media platforms (Facebook groups, WhatsApp groups, Instagram) to reach eligible participants. Informed consent was obtained from all participants electronically before participating in the study. The questionnaire is provided in the Supplementary Materials in both Turkish (Supplementary File S3) and English (Supplementary File S4).

Inclusion Criteria:

- Being a housewife aged 20–60 years.
- Married for at least 1 year.
- Performing a minimum of 1 h of daily housework.
- Experiencing wrist pain greater than 3/10 on the Numeric Pain Rating Scale (NPRS).
- Voluntarily agreed to participate and completed the online informed consent form.

Exclusion Criteria:

- Currently employed in any job (full-time or part-time).
- Wrist pain severity of less than 3/10 on the NPRS.
- History of upper-extremity injury or surgery within the last 12 months.
- Diagnosis of neurological and/or rheumatic diseases (self-reported).

### 2.3. Data Collection

**Figure 2:**
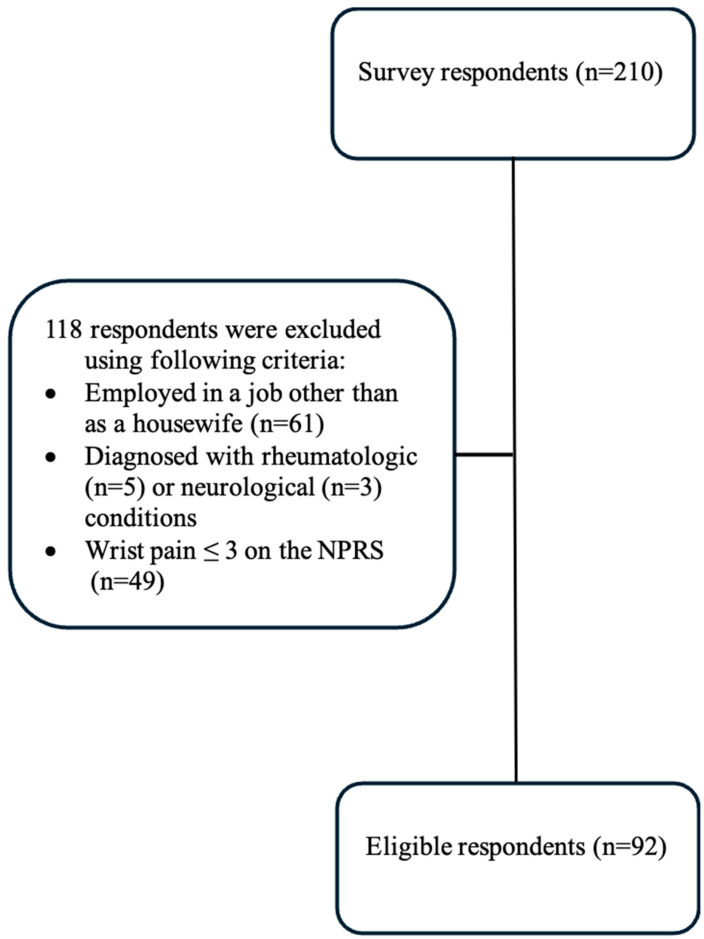
Sample selection flow diagram.

The data collection period was determined as 4 weeks, and the survey was closed to receive responses after 4 weeks. A total of 210 housewives participated in the study. Sixty-one women who were employed and forty-nine women with a wrist pain severity score of 3 cm or less were excluded from the study. In addition, a total of 8 participants diagnosed with rheumatological (n = 5) and neurological diseases (n = 3) were excluded (Figure 2).

In the first part of the online questionnaire, consisting of five sections, participants’ sociodemographic characteristics, duration of marriage and housework, and the number of children were questioned. The survey only included the daily duration of house labor; however, the type, intensity, and or the nature of the housework was not questioned. In addition to this information, wrist pain severity and fatigue severity were assessed using the NRS in the first part. In the second part, wrist pain and functionality levels of the participants were evaluated with the Turkish version of the Patient-Rated Wrist Evaluation Questionnaire (PRWE-T). In the third part, the Pain Catastrophizing Scale was used to determine the level of exaggerated negative orientation towards pain experiences. In the fourth part, the sense of confidence in performing functional activities despite pain was questioned with the Pain Self-Efficacy Questionnaire. In the last part, depression and anxiety levels of the participants were evaluated with the 4-question short version of the Patient Health Questionnaire.

### 2.4. Assessment of Fatigue and Wrist Pain

Fatigue and wrist pain were assessed using the Numeric Rating Scale (NRS), a tool considered reliable and valid for evaluating these parameters [24]. NRS is a simple and effective measurement tool for assessing a trait or attitude that is difficult to measure directly. It is scored between 0 and 10 [25]. The scale utilized in this study ranges from 0 to 10 points. Zero points indicate a state of absence of both pain and fatigue. Conversely, 10 points denote the presence of excruciating pain, profound fatigue [25]. Participants were asked to place a tick at the point on a scale of 0–10 that they thought best reflected their current situation.

### 2.5. Assessment of Wrist Pain and Functionality

Wrist pain and functionality levels were evaluated with the Turkish version of the Patient-Rated Wrist Evaluation Questionnaire (PRWE-T). The PRWE, developed by MacDermid et al. (1998), is a short, reliable, and valid questionnaire that comprehensively assesses patient-reported pain and dysfunction (ICC: 0.89–0.93, Cronbach’s Alpha: 0.92–0.98) [26]. The PRWE-T is a 15-item questionnaire (Cronbach’s Alpha: 0.88) that assesses the level of pain and functional disability in wrist problems. The validity and reliability of the Turkish version of the questionnaire were assessed by Öztürk et al. (2015) [27]. The first 5 items in the questionnaire are about pain, and the other 10 items are about function [26,27]. Each question is scored between 0 and 10, with 0 indicating no pain or difficulty and 10 indicating extreme pain and difficulty. While there are no cut-off values for categorizing, low total scores indicate that the pain and functionality level is good [26,27].

### 2.6. Determination of Pain Catastrophizing Level

The Pain Catastrophizing Scale (PCS) was used to determine the degree of exaggerated negative orientation towards participants’ pain experiences and catastrophic behaviors resulting from musculoskeletal pain [28]. The PCS is reported to be valid and reliable in the Turkish population (ICC: 0.95, Cronbach’s Alpha: 0.83) [29]. The 5-point Likert-type PCS, comprising a total of 13 items, is utilized to measure the presence of catastrophizing. Scores of 0 indicate the absence of catastrophizing, while scores of 4 indicate its presence. The total score ranges from 0 to 52, and an increase in the total score represents a higher level of pain catastrophizing; there are no cut-off values for categorizing [28,29].

### 2.7. Assessment of Pain Self-Efficacy Level

The degree of confidence of housewives in performing functional activities despite pain was assessed with the Pain Self-Efficacy Questionnaire (PSEQ). The Turkish validity and reliability study of PSEQ, developed by Nicholas et al. (2007) [30], was conducted by Sayılan et al. (2022) [31] (ICC: 0.90, Cronbach’s Alpha: 0.99). The scale consists of a total of 10 items. Each item has 7 response options, with 0 points designated as ‘I have no confidence in myself’ and 6 points designated as ‘I have complete confidence in myself’. The total score ranges from 0 to 60, with higher scores indicating a higher level of self-efficacy for functioning despite pain; there are no cut-off values for categorizing [30,31].

### 2.8. Assessment of Depression and Anxiety Levels

The 4-question short version of the Patient Health Questionnaire (PHQ-4) was used to evaluate depression and anxiety symptoms. The PHQ-4 is the shortest composite measurement tool that assesses both depression and anxiety symptoms, and the Cronbach’s alpha value of the questionnaire is reported as 0.85 [32]. The scale was adapted into Turkish by Demirci and Ekşi (2018) [33]. The questionnaire is scored for each question as 0: Never, 1: A few days, 2: More than half of the days, and 3: Almost every day. The first two items form the anxiety subscale, and a total score of 3 or higher is considered positive for anxiety. The last two items form the depression subscale, and a total score of 3 or higher is considered positive for depression [32,33].

### 2.9. Statistical Analysis

Data analyses were performed using the Statistical Package for Social Science for Windows Version 26.0 statistical software (SPSS Inc., an IBM Company, Chicago, IL, USA), and a significance level of a *p*-value less than 0.05 was accepted for all statistical analysis methods. The conformity of the variables to normal distribution was evaluated both visually (histograms, probability graphs) and analytically (Kolmogorov–Smirnov test). Qualitative variables are presented as numbers (n) and percentages (%), while quantitative variables are presented as the median (interquartile range) because the normality assumption was not met. The relationship between the demographic characteristics of the participants and the mean scale scores was analyzed using Spearman’s correlation test. The results of the correlation analysis were classified as follows: <0.20: “Poor”, 0.21–0.40: “Fair”, 0.41–0.60: “Moderate”, 0.61–0.80: “Good”, and >0.80: “Very Good” agreement [34]. To control for Type I error probability, Bonferroni correction for multiple correlations was determined by dividing the significance level by the number of tests performed [35]. A simple linear regression analysis was used to investigate the factors associated with the severity of wrist pain and dysfunction in housewives. Although simple linear regression analyses were conducted separately for each independent variable in the study to identify factors associated with wrist pain and functional impairment, a multivariate linear regression analysis was additionally performed to assess potential interactions and independent effects between the variables. In this context, the PRWE-T total score was included in the model as the dependent variable. The independent variables were identified as age, body mass index, daily housework duration, fatigue level, pain catastrophizing, pain self-efficacy, and psychological state. In the multivariate model, all variables were included in the analysis simultaneously, and the independent effect of each variable on the PRWE-T total score was assessed whilst controlling for the others. The model’s explanatory power was reported using the coefficient of determination (R^2^). The coefficients (B), standard errors (SE), standardized beta coefficients (β), and significance levels (*p*) for the regression analysis were presented.

## 3. Results

A total of 92 housewives were included in the study between November and December 2024. The mean age of the housewives included in the study was 42.21 ± 11.21 years, the mean duration of marriage was 20.78 ± 12.63 years, the mean number of children was 2.39 ± 1.34, and the mean daily housework time was 4.84 ± 2.26 h (Table 1).

Wrist pain severity, fatigue severity, pain catastrophizing, self-efficacy level, and depression and anxiety severity values of the participants are given in Table 2. According to the NPRS, the median values were 5.00 (4.00–6.75) for wrist pain and 7.00 (5.00–8.00) for fatigue severity. The median value of the PRWE-T total score evaluating the level of wrist pain and functionality was 42.25 (30.50–53.87), the median value of the PCS total score evaluating the level of pain catastrophizing was 18.50 (12.25–29.00), and the median value of the PSEQ evaluating the degree of confidence in performing functional activities despite pain was 42.00 (32.25–52.00). The median values of the depression and anxiety levels of the PHQ-4 sub-dimensions of the participants were equal, and the median value of the PHQ-4 total score was 4.00 (2.00–6.00) (Table 2).

Correlation analysis showed that as age increased, pain intensity (r = 0.261, *p* < 0.05/r = 0.256, *p* < 0.05), pain catastrophizing (r = 0.301, *p* < 0.001/r = 0.246, *p* < 0.05), and anxiety (r = 0.319, *p* < 0.001/r = 0.286, *p* < 0.001) symptoms also tended to be higher. Higher wrist pain scores were moderately associated with more functional limitations (r = 0.405, *p* < 0.001), increased emotional distress (r = 0.421, *p* < 0.001), and greater pain catastrophizing (r = 0.514, *p* < 0.001). Fatigue was linked to more pain (r = 0.255, *p* < 0.05) and dysfunction (r = 0.230, *p* < 0.05), as well as more depressive symptoms (r = 0.350, *p* < 0.001) and lower pain self-efficacy (r = −0.339, *p* < 0.001). The internal consistency of the PRWE-T subscales was strong, with pain and function scores showing strong associations with each other (r = 0.913, *p* < 0.001 and with the total score (r = 0.882, *p* < 0.001) (Table 3).

The presence of high levels of pain catastrophizing has been demonstrated to be associated with increased levels of wrist pain (r = 0.541), a greater limitation of function (r = 0.545), and elevated anxiety levels (r = 0.611), as well as elevated levels of overall psychological distress (r = 0.693) (*p* < 0.001). In contrast, housewives with higher levels of pain self-efficacy exhibit reduced pain and functional impairment (r = −0.232, *p* < 0.05), decreased pain catastrophizing (r = −0.233, *p* < 0.05), lower levels of anxiety (r = −0.437, *p* < 0.001), and psychological distress (r = −0.424, *p* < 0.001) (Table 4).

Higher depression and anxiety scores were moderately associated with increased wrist pain (r = 0.407, *p* < 0.001/r = 0.272, *p* < 0.001) and reduced function (r = 0.347, *p* < 0.001/r = 0.323, *p* < 0.001) as measured by the subscales of PRWE-T. Participants who reported higher overall psychological distress, as measured by the PHQ-4 total score, also tended to experience more wrist pain (r = 0.380, *p* < 0.001), greater wrist disability (r = 0.379, *p* < 0.001), and higher total PRWE-T scores (r = 0.418, *p* < 0.001). Depression and anxiety scores were also related to each other (r = 0.407, *p* < 0.001). Anxiety was additionally linked to more pain catastrophizing (r = 0.611, *p* < 0.001) and lower pain self-efficacy (r = 0.608, *p* < 0.001) (Table 4).

Simple linear regression analysis showed that higher fatigue severity, greater pain catastrophizing, and lower pain self-efficacy were significantly associated with increased wrist pain and reduced functionality among housewives. Additionally, higher depression and anxiety levels were associated with greater pain and poorer wrist function (Table 5).

The multivariable regression analysis showed that higher fatigue and higher pain catastrophizing were associated with higher PRWE-T total scores. In contrast, age, BMI, daily duration of housework, pain self-efficacy, and PHQ-4 total score did not remain significant in the adjusted model. Independent parameters explained 41.5% of the variance in the PRWE-T total score (Table 6).

## 4. Discussion

The objective of the present study was to evaluate the severity of wrist pain and disability in housewives and to identify the psychosocial and symptom-related factors associated with these outcomes, including fatigue, pain catastrophizing, pain self-efficacy, depression, and anxiety. To the best of our knowledge, this is the first comprehensive study in Turkey investigating specifically wrist symptoms and related parameters in housewives. Although numerous studies have been conducted internationally, the definition of homemaking women varies across cultural contexts, highlighting the contribution of the present study.

Our cross-sectional study demonstrated that housewives experiencing wrist pain exhibited elevated levels of wrist-related pain severity and functional impairment. In addition, fatigue severity was high in this population, accompanied by increased levels of anxiety, depression, and pain catastrophizing. However, the pain self-efficacy levels remained high. Correlation analyses revealed a significant positive relationship between age and pain, pain catastrophizing, anxiety, and depression. In bivariate analyses, higher fatigue, pain catastrophizing, lower pain self-efficacy, and higher depression and anxiety scores were associated with greater wrist pain and disability. In the multivariable model, pain catastrophizing and fatigue remained significantly associated with the PRWE-T total score.

Researchers have previously examined musculoskeletal complaints in housewives, which demonstrated that heavy lifting and repetitive, forceful movements associated with housework, childcare, and psychosocial factors, including domestic stress and family-related responsibilities, have a negative impact on musculoskeletal health, specifically in developing countries similar to Turkey [15,19,36]. Our results showed high PRWE-T total, pain, and function scores among Turkish housewives, possibly reflecting the demanding nature of household activities and the associated upper-extremity workload [37]. The literature has shown that repetitive minor hand and wrist movements, even with minimal force, can result in common musculoskeletal impairments [38]. The specific nature of the housework performed influences both the pattern of repetitive joint loading and the mechanisms through which musculoskeletal disorders develop. However, because neither the type (e.g., wringing out cloths, washing by hand, or handling heavy utensils) nor the details of the housework performed nor any physical/biomechanical measurements were assessed due to the online data collection method, it is not possible to draw definitive conclusions regarding these relationships.

The comprehensive assessment in the present study included the daily duration of household chores. Hafeez et al. (2024) also examined this parameter and found that engaging in household chores for 8 h or more was linked to increased pain levels [36]. In contrast, the present study did not reveal a significant relationship between the duration of daily household chores and pain, disability, or pain-related behaviors. This may be attributed to the relatively shorter average duration of daily household chores in the present sample, which was 5 h, considerably lower than those reported in other studies. This lower duration could have limited the ability to detect significant correlations in the present analysis. Moreover, it is possible that the content and nature of household tasks, rather than their total duration, play a more critical role in the development of wrist pain, which we may have been unable to capture, as our study did not assess task-specific activities.

Pain catastrophizing has been identified as an important psychological factor in the pain experience and has been shown to be associated with increased pain, disability, depression, and anxiety, as well as poor treatment outcomes in chronic pain conditions [39,40]. In line with this evidence, the current study identified high levels of pain catastrophizing among housewives with wrist pain [41,42]. Increased catastrophizing scores in housewives can be explained by the positive impact of stronger social relationships and overall well-being observed among working individuals [43]. Multivariable regression analyses have shown that pain catastrophizing and fatigue levels were significant independent correlates of wrist pain and functionality. Furthermore, correlation analyses revealed that increased pain catastrophizing was associated with wrist pain, depression, anxiety, and fatigue. Specifically, studies demonstrate that depression is more prevalent in non-working women and is significantly associated with pain and quality of life [44,45].

Additionally, over 70% of the housewives in the present study exhibited low educational levels. This finding aligns with previous literature indicating a relationship between educational level and pain catastrophizing [46]. However, participant recruitment through online convenience sampling may have selectively favored women who had sufficient access to digital devices and internet connectivity, as well as the ability to complete an online survey. Moreover, lower educational attainment may also have influenced how some participants read, understood, and interpreted the survey items. To address these issues, future studies should consider face-to-face research designs that allow for a more comprehensive and representative assessment of housewives.

Fatigue was found to be another critical factor in housewives with wrist pain. The literature supports this finding, indicating that fatigue can impair functional performance and proprioception in the wrist joint [47]. The relationship between catastrophic behavior and fatigue has been previously examined in diseases involving chronic pain, and although the underlying mechanism is not yet fully understood, it has been reported as an explanatory factor for fatigue [48]. However, fatigue was assessed using a single-item NRS, which does not capture its multidimensional nature and may limit the interpretation of these relationships. Another pain behavior investigated in the present study was pain self-efficacy. It has been shown that high pain self-efficacy prevents the development of fear avoidance behaviors, helps to maintain the functional status of the individual, and positively affects recovery by increasing participation in rehabilitation [49].

The present results demonstrated that pain self-efficacy in housewives with wrist pain was associated with higher pain and disability, a finding that aligns with Hiraga et al. (2021)’s research, where they also identified a significant relationship between wrist pain and pain self-efficacy using the PRWE and PSEQ [50]. The correlation analysis showed that, in housewives experiencing wrist pain, higher pain self-efficacy was associated with lower levels of depression and anxiety, highlighting the positive effects of pain self-efficacy. Numerous studies in the literature have reported lower levels of pain self-efficacy among housewives and non-working individuals [51,52]. In the present study, the mean PSEQ score was 42. Although no specific cut-off value for this scale was established, this score is higher than those reported in other studies involving housewives with chronic pain. This finding may be attributed to two factors: the younger average age of the women included in the present sample, or the possibility that these individuals have developed more effective coping strategies over time due to the sociocultural responsibilities they bear at home.

Correlation analyses showed that increased age was associated with both wrist pain and pain catastrophizing. These associations may be explained by the cumulative physical demands and social roles traditionally assumed by housewives over extended periods. Another important factor questioned was the number of children. Previous studies have suggested that raising multiple children may have a detrimental impact on musculoskeletal health. For example, Kaur et al. (2024) reported that 50.3% of the women who have at least four children develop musculoskeletal disorders [9]. Similarly, another researcher has shown that the number of children has a significant negative impact on musculoskeletal health [15]. However, this study did not identify a significant relationship between the number of children and wrist pain or wrist pain-related dysfunction. This finding may be attributed to the relatively low mean number of children (2.39 ± 1.34) among the housewives included in the study, potentially reducing the impact of childcare.

This study’s strengths include its comprehensive examination of housewives’ sociodemographic characteristics and daily life factors using proven, reliable assessment methods. Furthermore, to the best of our knowledge, this is the first study to specifically examine wrist pain and related factors in Turkish housewives. However, the present study also has several limitations. First, due to its cross-sectional design, it was not possible to directly determine causality via the correlation and regression analyses. Since this study was conducted via an open online survey, a convenience sampling method was used, and this may have led to selection bias. In addition to the low response rates typically observed in web-based surveys, women with greater access to digital devices and digital literacy may have been overrepresented in the sample, while women with lower literacy levels may have been underrepresented. Therefore, the generalizability of these findings is limited. Another limitation is that, since wrist pain was intended to be examined as a general pain complaint, it was evaluated without being classified according to duration of symptoms, severity, side, or suspected diagnosis. To ensure that the survey was shorter and more feasible, and to increase the participation rate, fatigue was assessed using a single-item NRS rather than a comprehensive multidimensional scale, which may constitute a methodological limitation. As mentioned above, housewives represent a population that is rarely studied, which made it challenging to compare the present findings with similar studies in the literature. Additionally, the nature of housework may vary across regional and cultural contexts. Therefore, it is important to acknowledge that this study was conducted among housewives living in Turkey, and each woman may have a unique perspective on domestic work and adopt distinct roles and responsibilities. Additionally, the domestic workload was assessed solely through participants’ self-reported daily hours, without specifying task types or incorporating any objective measurement of biomechanical load. This limits the objective demonstration of the relationship between wrist pain and biomechanical stress and should be addressed in future studies through more comprehensive and objective assessment methods. Accordingly, future studies with larger samples, objective and multidimensional assessments, and longitudinal designs are needed to better clarify these relationships.

## 5. Conclusions

The aim of this study was to evaluate the severity of wrist pain and wrist-related disability in housewives and to identify the psychosocial and symptom-related factors associated with these outcomes. Our findings indicate that catastrophizing, pain self-efficacy, anxiety, depression, and age may be associated with wrist pain severity and related disability. The present findings indicate the high severity of wrist symptoms in housewives, an understudied population. Furthermore, these symptoms showed associations with psychological and demographic parameters. Future research should include longitudinal or interventional studies to clarify potential causal relationships and determine whether ergonomic or psychosocial approaches contribute to symptom management.

## Figures and Tables

**Table 1:** Demographic characteristics of participants (n = 92).

		Median (Q1–Q3)	Mean ± SD
**Age (years)**		43.00 (31.25–52.00)	42.21 ± 11.21
**Weight (kg)**		70.00 (65.00–76.00)	72.18 ± 13.94
**Height (cm)**		162.00 (160.00–167.00)	162.51 ± 5.84
**BMI (kg/m^2^)**		26.52 (24.45–29.40)	27.34 ± 5.23
**Dominant Upper Extremity (n/%)**	**Right**	79/ %85.9	
	**Left**	13/ %14.1	
**Education Level (n/ %)**	Elementary school	24/ %26.1	
	Middle school	14/ %15.2	
	High school	28/ %30.4	
	Associate degree	16/ %17.4	
	Bachelor’s degree	9/ %9.8	
	Graduate school	1/ %1.1	
**Marriage Duration (years)**		23.00 (8.25–32.00)	20.78 ± 12.63
**Number of children**		2.00 (1.00–3.00)	2.39 ± 1.34
**Daily duration of house labor (hours)**		5.00 (3.00–6.00)	4.84 ± 2.26

Data was given as the median (Interquartile range) and percentiles. kg, kilogram; cm, centimeter; SD: standard deviation.

**Table 2:** Mean scores of the evaluated parameters in participants (n = 92).

	Median (Q1–Q3)	Mean ± SD
**PRWE-T-Pain**	25.00 (20.25–33.0)	26.69 ± 9.11
**PRWE-T-Function**	35.00 (17.75–47.00)	34.36 ± 19.95
**PRWE-T-Total**	42.25 (30.50–53.87)	43.88 ± 17.30
**PCS**	18.50 (12.25–29.00)	20.21 ± 12.23
**PSEQ**	42.00 (32.25–52.00)	40.70 ± 13.32
**PHQ-4 Depression**	2.00 (1.00–3.00)	2.17 ±1.44
**PHQ-4 Anxiety**	2.00 (1.00–3.00)	2.05 ± 1.56
**PHQ-4 Total**	4.00 (2.00–6.00)	4.22 ± 2.69
**Wrist pain-NRS**	5.00 (4.00–6.75)	5.21 ± 1.85
**Fatigue-NRS**	7.00 (5.00–8.00)	6.73 ± 2.08

Data was given as the median (Interquartile range). PRWE-T: Patient-Rated Wrist Evaluation-Turkish; PCS: Pain Catastrophizing Scale; PSEQ: Pain Self-Efficacy Questionnaire; PHQ-4: Patient Health Questionnaire-4; NRS: Numeric Rating Scale; SD: standard deviation.

**Table 3:** Correlations between demographic characteristics and mean scale scores of the participants.

	PRWE-T-Pain	PRWE-T-Function	PRWE-T-Total	PCS	PSEQ	PHQ-4 Depression	PHQ-4 Anxiety	PHQ-4 Total
**Age**	0.261 * **^a^**	0.051	0.161	**0.301** * **^a^**	−0.144	0.171	**0.319** * **^a^**	**0.275** * **^a^**
**BMI**	0.195	0.031	0.120	0.199	−0.117	0.158	0.109	0.134
**Number of children**	0.111	−0.075	0.008	0.031	−0.067	0.014	0.086	0.064
**Daily duration of house labor**	0.161	0.118	0.172	0.183	−0.074	0.165	0.179	0.189
**Wrist pain-NRS**	**0.599** * **^a^**	**0.405** * **^a^**	**0.536** * **^a^**	**0.514** * **^a^**	−0.146	**0.421** * **^a^**	**0.368** * **^a^**	**0.454** * **^a^**
**Fatigue-NRS**	0.255 * **^a^**	0.230 * **^a^**	0.263 * **^a^**	**0.331** * **^a^**	**−0.339** * **^a^**	**0.350** * **^a^**	0.185	**0.298** * **^a^**

PRWE-T: Patient Rated Wrist Evaluation-Turkish; PCS: Pain Catastrophizing Scale; PSEQ: Pain Self-Efficacy Questionnaire; PHQ-4: Patient Health Questionnaire-4; NRS: Numeric Rating Scale; * ^a^: Following Bonferroni correction for multiple correlations, statistical significance was set at *p* = 0.05/56 = 0.00089. Bold values indicate statistically significant results.

**Table 4:** Correlations between mean scale scores of the participants.

	PRWE-T-Pain	PRWE-T-Function	PRWE-T-Total	PCS	PSEQ	PHQ-4 Depression	PHQ-4 Anxiety	PHQ-4 Total
**PRWE-T-Pain**	1.000	**0.634** * **^a^**	**0.882** * **^a^**	**0.541** * **^a^**	−0.187	**0.407** * **^a^**	**0.272** * **^a^**	**0.380** * **^a^**
**PRWE-T Function**		1.000	**0.913** * **^a^**	**0.545** * **^a^**	−0.233 * ^a^	**0.347** * **^a^**	**0.323** * **^a^**	**0.379** * **^a^**
**PRWE-T Total**			1.000	**0.598** * **^a^**	−0.232 * ^a^	**0.418** * **^a^**	**0.324** * **^a^**	**0.418** * **^a^**
**PCS**				1.000	**−0.437** * **^a^**	**0.604** * **^a^**	**0.611** * **^a^**	**0.693** * **^a^**
**PSEQ**					1.000	**−0.502** * **^a^**	**−0.424** * **^a^**	**−0.486** * **^a^**
**PHQ-4 Depression**						1.000	**0.608** * **^a^**	**0.890** * **^a^**
**PHQ-4 Anxiety**							1.000	**0.888** * **^a^**
**PHQ-4**								1.000

PRWE-T: Patient Rated Wrist Evaluation-Turkish; PCS: Pain Catastrophizing Scale; PSEQ: Pain Self-Efficacy Questionnaire; PHQ-4: Patient Health Questionnaire-4; NPRS: Numeric Pain Rating Scale; * ^a^: Following Bonferroni correction for multiple correlations, statistical significance was set at *p* = 0.05/28 = 0.00179. Bold values indicate statistically significant results.

**Table 5:** Simple linear regression.

	PRWE-T-Pain			PRWE-T-Function			PRWE-T-Total		
	B	R^2^	*p*	B	R^2^	*p*	B	R^2^	*p*
**BMI**	0.454	0.068	**0.012**	0.412	0.012	0.304	0.660	0.040	0.056
**Number of children**	0.555	0.007	0.439	−1.045	0.005	0.506	0.032	0.000	0.981
**Daily duration of house labor**	0.636	0.025	0.132	0.855	0.009	0.357	1.064	0.019	0.185
**Wrist pain-NRS**	3.102	0.399	**<0.001**	4.341	0.163	**<0.001**	5.273	0.320	**<0.001**
**Fatigue-NRS**	1.353	0.095	**0.003**	2.641	0.076	**0.008**	2.673	0.103	**0.002**
**PRWE-T-Pain**	-	-	-	1.408	0.413	**<0.001**	1.704	0.805	**<0.001**
**PRWE-T-Function**	0.294	0.413	**<0.001**	-	-	-	0.794	0.837	**<0.001**
**PRWE-T-Total**	0.472	0.805	**<0.001**	1.055	0.837	**<0.001**	-	-	-
**PCS**	0.425	0.326	**<0.001**	0.910	0.311	**<0.001**	0.880	0.387	**<0.001**
**PSEQ**	−0.159	0.054	**0.026**	−0.329	0.048	**0.035**	−0.324	0.062	**0.017**
**PHQ-4-Depression**	2.442	0.151	**<0.001**	4.697	0.116	**<0.001**	4.790	0.161	**<0.001**
**PHQ-4-Anxiety**	1.753	0.091	**0.004**	3.880	0.093	**0.003**	3.693	0.111	**0.001**
**PHQ-Total**	1.295	0.147	**<0.001**	2.661	0.129	**<0.001**	2.625	0.167	**<0.001**

PRWE-T: Patient Rated Wrist Evaluation-Turkish; PCS: Pain Catastrophizing Scale; PSEQ: Pain Self-Efficacy Questionnaire; PHQ-4: Patient Health Questionnaire-4; NRS: Numeric Rating Scale. Bold values indicate statistically significant results.

**Table 6:** Multivariable regression of factors associated with PRWE-T total score.

	B	SE	β	*p*	R^2^
**Age**	−0.074	0.157	−0.048	0.640	0.415
**BMI**	0.401	0.314	0.121	0.206	
**Daily duration of house labor**	0.054	0.684	0.007	0.937	
**Fatigue-NRS**	1.145	0.837	0.138	0.042 *	
**PCS**	0.879	0.176	0.621	<0.001 **	
**PSEQ**	0.085	0.131	0.066	0.517	
**PHQ-4 Total**	−0.257	0.790	−0.040	0.745	

BMI, Body Mass Index; NRS, Numeric Rating Scale; PCS, Pain Catastrophizing Scale; PSEQ, Pain Self-Efficacy Questionnaire; PHQ, Patient Health Questionnaire; *, *p* < 0.05; **, *p* < 0.001.

## Data Availability

The datasets produced or scrutinized in the present study can be obtained upon reasonable request from the corresponding author. Raw data are not publicly available due to confidentiality commitments stated in the informed consent form.

## Supplementary Materials

The following supporting information can be downloaded at: https://www.mdpi.com/article/10.3390/healthcare14091162/s1, Supplementary File S1: Checklist for Reporting Results of Internet E-Surveys (CHERRIES); Supplementary File S2: STROBE Statement—Checklist of items that should be included in reports of cross-sectional studies; Supplementary File S3: Turkish Online Questionnaire Form; Supplementary File S4: English Online Questionnaire Form.
