# Supplementary material for: Psychosocial and Sociodemographic Factors Associated with Wrist Pain Severity and Dysfunction in Turkish Housewives: A Web-Based Cross-Sectional Survey
Source: Healthcare (Basel). 2026 Apr 26;14(9):1162. doi: 10.3390/healthcare14091162 (PMC13163648; doi:10.3390/healthcare14091162)
Supplement: Supplementary file 1 [file healthcare-14-01162-s001.zip › S3.pdf]

El Bileği Ağrısı Olan Ev Hanımlarında Ağrı ve Disfonksiyon Şiddetinin İncelenmesi ve İlişkili Faktörlerin Belirlenmesi

Bu çalışma Toros Üniversitesi Sağlık Bilimleri Fakültesi Fizyoterapi ve Rehabilitasyon Bölümü Araştırma Görevlisi Uzm. Fzt. Ece EKİCİ tarafından yürütülmektedir. "**El Bileği Ağrısı Olan Ev Hanımlarında Ağrı ve Disfonksiyon Şiddetinin İncelenmesi ve İlişkili Faktörlerin Belirlenmesi**" isimli çalışmada yer almak üzere davet edilmiş bulunmaktasınız. Size 15-20 dakikalık süre içerisinde el bileği ağrınızı ve ağrınızın günlük yaşam aktivitelerine etkisini sorgulayan anketler uygulanacaktır. Anketten elde edilen yanıtlar tamamen gizli kalacak, sadece bilimsel amaçlarla kullanılacaktır. Çalışma sırasında sizden herhangi bir ücret talep edilmeyecek, yanıtlamayı kabul ettiğiniz durumda size herhangi bir ücret ödenmeyecektir.
Çalışmamıza katılmayı kabul ettiğiniz için çok teşekkür ederiz.

* Zorunlu soruyu belirtir

1. Yaşınızı belirtiniz. *

2. Vücut ağırlığınızı belirtiniz (kg). *

3. Boy uzunluğunuzu belirtiniz (cm). *

4. Ev hanımlığı dışında profesyonel bir işte düzenli olarak çalışıyor musunuz? *

Yalnızca bir şıkkı işaretleyin.

- Evet
- Hayır

5. Eğitim düzeyinizi belirtiniz *

Yalnızca bir şıkkı işaretleyin.

- İlkokul
- Ortaokul
- Lise
- Önlisans
- Lisans
- Lisansüstü

6. Herhangi bir omuz, dirsek, el, el bileği ile ilgili uzman doktordan alınmış bir tanınız var mı? *

Yalnızca bir şıkkı işaretleyin.

- Evet
- Hayır

7. Yukarıdaki soruya cevabınız "Evet" ise hastalığınızı belirtiniz.

8. Bir önceki soruda belirttiğiniz hastalığa yönelik bir ilaç kullanıyor musunuz?

Yalnızca bir şıkkı işaretleyin.

- Evet
- Hayır

9. Yukarıdaki soruya cevabınız "Evet ise kullandığınız ilaçları yazınız.

10. Kaç yıldır evlisiniz? *

____

11. Kaç çocuğunuz var? *

____

12. Günlük ortalama kaç saat ev iş yapıyorsunuz? (temizlik yapmak, yemek yapmak, ütü yapmak, bulaşık yıkamak...vb.) *

____

13. Günlük yaşamınızda daha sık hangi elinizi kullanırsınız? (Dominant elinizi belirtiniz.) *

Yalnızca bir şıkkı işaretleyin.

- [ ] Sağ
- [ ] Sol

14. El bileği ağrınızın şiddetini aşağıda belirtiniz. *

Yalnızca bir şıkkı işaretleyin.

	0	1	2	3	4	5	6	7	8	9	10	
Hiç	◯	◯	◯	◯	◯	◯	◯	◯	◯	◯	◯	Ağrım dayanılmaz derecede

15. Gün içerisinde yaşadığınız yorgunluk şiddetini belirtiniz. *

Yalnızca bir şıkkı işaretleyin.

	0	1	2	3	4	5	6	7	8	9	10	
Hiç	◯	◯	◯	◯	◯	◯	◯	◯	◯	◯	◯	Yorgunluğum dayanılmaz derecede

HASTA BAZLI EL BİLEĞİ DEĞERLENDİRME ANKETİ

Son bir haftadaki el bileğinizle ilgili ortalama şikayetlerinizi 0-10 dereceli bir skala ile tanımlayacaksınız. Eğer son 1 hafta içinde aktiviteyi yapmadıysanız lütfen beklediğiniz zorlanma veya ağrı derecesini belirtiniz.

AĞRI

16. İstirahatte (Dinlenmede) *

Yalnızca bir şıkkı işaretleyin.

	0	1	2	3	4	5	6	7	8	9	10	
Ağrı	◯	◯	◯	◯	◯	◯	◯	◯	◯	◯	◯	Dayanılmaz ağrı

17. Tekrarlı el bilek hareketlerini içeren bir iş yapıldığında *

Yalnızca bir şıkkı işaretleyin.

	0	1	2	3	4	5	6	7	8	9	10	
Ağrı	◯	◯	◯	◯	◯	◯	◯	◯	◯	◯	◯	Dayanılmaz ağrı

18. Ağır bir nesneyi kaldırırken *

Yalnızca bir şıkkı işaretleyin.

	0	1	2	3	4	5	6	7	8	9	10	
Ağrı	◯	◯	◯	◯	◯	◯	◯	◯	◯	◯	◯	Dayanılmaz ağrı

19. En kötü olduğu zaman *

Yalnızca bir şıkkı işaretleyin.

	0	1	2	3	4	5	6	7	8	9	10	
Ağrı	◯	◯	◯	◯	◯	◯	◯	◯	◯	◯	◯	Dayanılmaz ağrı

20. Hangi sıklıkta ağrınız var? *

Yalnızca bir şıkkı işaretleyin.

	0	1	2	3	4	5	6	7	8	9	10	
Hiçb	◯	◯	◯	◯	◯	◯	◯	◯	◯	◯	◯	Her zaman

FONKSİYON

A. Spesifik Aktiviteler

21. Etkilenen elimi kullanarak kapı kolu çevirmek *

Yalnızca bir şıkkı işaretleyin.

	0	1	2	3	4	5	6	7	8	9	10	
Hiç :	◯	◯	◯	◯	◯	◯	◯	◯	◯	◯	◯	Yapmak mümkün değil

22. Etkilenen elimle bıçak kullanarak et doğramak *

Yalnızca bir şıkkı işaretleyin.

	0	1	2	3	4	5	6	7	8	9	10	
Hiç :	◯	◯	◯	◯	◯	◯	◯	◯	◯	◯	◯	Yapmak mümkün değil

23. Gömlek düğmesi iliklemek *

Yalnızca bir şıkkı işaretleyin.

	0	1	2	3	4	5	6	7	8	9	10	
Hiç :	◯	◯	◯	◯	◯	◯	◯	◯	◯	◯	◯	Yapmak mümkün değil

24. Etkilenen elimden destek alarak sandalyeden kalkmak *

Yalnızca bir şıkkı işaretleyin.

	0	1	2	3	4	5	6	7	8	9	10	
Hiç :	◯	◯	◯	◯	◯	◯	◯	◯	◯	◯	◯	Yapmak mümkün değil

25. Etkilenen elimle 4,5 kilogram ağırlık taşımak *

Yalnızca bir şıkkı işaretleyin.

	0	1	2	3	4	5	6	7	8	9	10	
Hiç :	◯	◯	◯	◯	◯	◯	◯	◯	◯	◯	◯	Yapmak mümkün değil

26. Etkilenen elimle tuvalet kağıdı kullanmak *

Yalnızca bir şıkkı işaretleyin.

	0	1	2	3	4	5	6	7	8	9	10	
Hiç :	◯	◯	◯	◯	◯	◯	◯	◯	◯	◯	◯	Yapmak mümkün değil

B. Günlük Aktiviteler

27. Kişisel bakım aktiviteleri (Giyinme,duş) *

Yalnızca bir şıkkı işaretleyin.

	0	1	2	3	4	5	6	7	8	9	10	
Hiç :	◯	◯	◯	◯	◯	◯	◯	◯	◯	◯	◯	Yapmak mümkün değil

28. Ev işleri (Temizlik, bakım) *

Yalnızca bir şıkkı işaretleyin.

	0	1	2	3	4	5	6	7	8	9	10	
Hiç :	◯	◯	◯	◯	◯	◯	◯	◯	◯	◯	◯	Yapmak mümkün değil

29. İş (Mesleğiniz veya günlük çalışmanız) *

Yalnızca bir şıkkı işaretleyin.

	0	1	2	3	4	5	6	7	8	9	10	
Hiç :	◯	◯	◯	◯	◯	◯	◯	◯	◯	◯	◯	Yapmak mümkün değil

30. Boş zaman aktiviteleri *

Yalnızca bir şıkkı işaretleyin.

	0	1	2	3	4	5	6	7	8	9	10	
Hiç :	◯	◯	◯	◯	◯	◯	◯	◯	◯	◯	◯	Yapmak mümkün değil

AĞRI FELAKETLEŞTİRME ÖLÇEĞİ

Hemen herkes hayatının bir bölümünde ağrı yaşamıştır (baş ağrısı, diş ağrısı, eklem ağrısı, kas ağrısı gibi). Biz ağrı yaşadığınız zamanlardaki duygu ve düşüncelerinizle ilgileniyoruz. Aşağıda ağrıyla ilişkili farklı duygu ve düşünceleri tanımlayan 13 durum yer almaktadır. Bu ölçeği kullanarak, ağrı yaşadığınız anlardaki duygu ve düşüncelerinizin derecesini belirtiniz.

31. Ağrının sona erip ermeyeceği konusunda sürekli endişelenirim. *

Yalnızca bir şıkkı işaretleyin.

- Hiç Yok
- Hafif Derece
- Orta Derece
- Büyük Ölçüde
- Her zaman

32. Ağrı nedeniyle devam edemeyeceğimi hissederim *

Yalnızca bir şıkkı işaretleyin.

- Hiç Yok
- Hafif Derece
- Orta Derece
- Büyük Ölçüde
- Her zaman

33. Ağrının korkunç olduğunu ve asla düzelmeyeceğini düşünürüm *

Yalnızca bir şıkkı işaretleyin.

- Hiç Yok
- Hafif Derece
- Orta Derece
- Büyük Ölçüde
- Her zaman

34. Ağrı berbat bir şeydir ve beni bunalttığını hissederim *

Yalnızca bir şıkkı işaretleyin.

- Hiç Yok
- Hafif Derece
- Orta Derece
- Büyük Ölçüde
- Her zaman

35. Ağrıya daha fazla dayanamayacağımı hissederim *

Yalnızca bir şıkkı işaretleyin.

- Hiç Yok
- Hafif Derece
- Orta Derece
- Büyük Ölçüde
- Her zaman

36. Ağrının kötüleşeceğinden korkarım *

Yalnızca bir şıkkı işaretleyin.

- Hiç Yok
- Hafif Derece
- Orta Derece
- Büyük Ölçüde
- Her zaman

37. Sürekli olarak başka ağrılı durumları düşünürüm *

Yalnızca bir şıkkı işaretleyin.

- Hiç Yok
- Hafif Derece
- Orta Derece
- Büyük Ölçüde
- Her zaman

38. Endişeli biçimde ağrının geçmesini dilerim *

Yalnızca bir şıkkı işaretleyin.

- Hiç Yok
- Hafif Derece
- Orta Derece
- Büyük Ölçüde
- Her zaman

39. Ağrıyı kafamdan atamıyorum *

Yalnızca bir şıkkı işaretleyin.

- Hiç Yok
- Hafif Derece
- Orta Derece
- Büyük Ölçüde
- Her zaman

40. Sürekli olarak ağrının canımı ne kadar yaktığını düşünürüm *

Yalnızca bir şıkkı işaretleyin.

- Hiç Yok
- Hafif Derece
- Orta Derece
- Büyük Ölçüde
- Her zaman

41. Ağrının geçmesini beklemenin ne kadar zor olduğunu düşünüp dururum *

Yalnızca bir şıkkı işaretleyin.

- Hiç Yok
- Hafif Derece
- Orta Derece
- Büyük Ölçüde
- Her zaman

42. Ağrının şiddetini azaltmak için yapabileceğim hiçbir şey yok *

Yalnızca bir şıkkı işaretleyin.

- Hiç Yok
- Hafif Derece
- Orta Derece
- Büyük Ölçüde
- Her zaman

43. Ağrının ciddi bir sorunla ilgili olup olmadığını merak ederim *

Yalnızca bir şıkkı işaretleyin.

- Hiç Yok
- Hafif Derece
- Orta Derece
- Büyük Ölçüde
- Her zaman

AĞRI ÖZ-YETERLİLİK ANKETİ

Ağrılarınıza rağmen, şu anda aşağıdakileri yapabileceğinize ne kadar güvendiğinizi derecelendiriniz.

44. Ağrıma rağmen, bir şeylerden keyif alabilirim. *

Yalnızca bir şıkkı işaretleyin.

	0	1	2	3	4	5	6	
Ken(	◯	◯	◯	◯	◯	◯	◯	Kendime tamamen güveniyorum

45. Ağrıma rağmen, ev işlerinin çoğunu (evi toplamak, bulaşık yıkamak vb.) yapabilirim. *

Yalnızca bir şıkkı işaretleyin.

	0	1	2	3	4	5	6	
Ken(	◯	◯	◯	◯	◯	◯	◯	Kendime tamamen güveniyorum

46. Ağrıma rağmen, arkadaşlarımla ya da ailemle eskiden olduğu kadar sık sosyalleşebilirim. *

Yalnızca bir şıkkı işaretleyin.

	0	1	2	3	4	5	6	
Ken(	◯	◯	◯	◯	◯	◯	◯	Kendime tamamen güveniyorum

47. Çoğu durumda ağrılarımla başa çıkabilirim. *

Yalnızca bir şıkkı işaretleyin.

	0	1	2	3	4	5	6	
Ken(	◯	◯	◯	◯	◯	◯	◯	Kendime tamamen güveniyorum

48. Ağrıma rağmen, çeşitli işler yapabilirim. (ev işi veya meslek işleri vb) *

Yalnızca bir şıkkı işaretleyin.

	0	1	2	3	4	5	6	
Ken(	◯	◯	◯	◯	◯	◯	◯	Kendime tamamen güveniyorum

49. Ağrıma rağmen, hobi ve boş zaman aktiviteleri gibi yapmaktan hoşlandığım birçok şeyi hala yapabilirim. *

Yalnızca bir şıkkı işaretleyin.

	0	1	2	3	4	5	6	
Ken(	◯	◯	◯	◯	◯	◯	◯	Kendime tamamen güveniyorum

50. İlaç tedavisi olmadan ağrımla başa çıkabilirim. *

Yalnızca bir şıkkı işaretleyin.

	0	1	2	3	4	5	6	
Ken	◯	◯	◯	◯	◯	◯	◯	Kendime tamamen güveniyorum

51. Ağrıma rağmen, hala hayatımdaki birçok hedefe ulaşabilirim. *

Yalnızca bir şıkkı işaretleyin.

	0	1	2	3	4	5	6	
Ken	◯	◯	◯	◯	◯	◯	◯	Kendime tamamen güveniyorum

52. Ağrıma rağmen, normal bir yaşam tarzı sürdürebilirim. *

Yalnızca bir şıkkı işaretleyin.

	0	1	2	3	4	5	6	
Ken	◯	◯	◯	◯	◯	◯	◯	Kendime tamamen güveniyorum

53. Ağrıma rağmen, giderek daha aktif olabilirim. *

Yalnızca bir şıkkı işaretleyin.

	0	1	2	3	4	5	6	
Ken	◯	◯	◯	◯	◯	◯	◯	Kendime tamamen güveniyorum

HASTA SAĞLIK ANKETİ-4

Son 2 hafta boyunca, aşağıdaki problemlerden dolayı ne sıklıkla sorun yaşadınız? Lütfen cevabınızı belirten kutuya bir onay işareti koyunuz.

54. Endişeli, huzursuz ya da tedirgin hissetmek *

Yalnızca bir şıkkı işaretleyin.

- Hiç
- Birkaç gün
- Günlerin yarısından fazlasında
- Neredeyse her gün

55. Tasalanmayı durduramamak ya da kontrol edememek *

Yalnızca bir şıkkı işaretleyin.

- Hiç
- Birkaç gün
- Günlerin yarısından fazlasında
- Neredeyse her gün

56. Bir şeyler yapmaya az ilgi duymak ya da yapmaktan az zevk almak *

Yalnızca bir şıkkı işaretleyin.

- Hiç
- Birkaç gün
- Günlerin yarısından fazlasında
- Neredeyse her gün

57. Moralsiz, depresif veya umutsuz hissetmek *

Yalnızca bir şıkkı işaretleyin.

- Hiç
- Birkaç gün
- Günlerin yarısından fazlasında
- Neredeyse her gün

Anketimiz tamamlanmıştır...Katılımınız ve sabrınız için çok teşekkür ederiz...

Bu içerik Google tarafından oluşturulmamış veya onaylanmamıştır.

Google Formlar
