# Supplementary material for: Psychosocial and Sociodemographic Factors Associated with Wrist Pain Severity and Dysfunction in Turkish Housewives: A Web-Based Cross-Sectional Survey
Source: Healthcare (Basel). 2026 Apr 26;14(9):1162. doi: 10.3390/healthcare14091162 (PMC13163648; doi:10.3390/healthcare14091162)
Supplement: Supplementary file 1 [file healthcare-14-01162-s001.zip › S4.pdf]

Psychosocial and Sociodemographic Correlates of Wrist Pain and Dysfunction in Turkish Housewives: A Cross-Sectional Study

This study is being conducted by Research Assistant Specialist Physiotherapist Ece EKİCİ from the Department of Physiotherapy and Rehabilitation, Faculty of Health Sciences, Toros University. You have been invited to participate in the study titled "Investigation of Pain and Dysfunction Severity and Determination of Associated Factors in Housewives with Wrist Pain." Questionnaires that ask about your wrist pain and the effect of your pain on activities of daily living will be applied and will take approximately 15–20 minutes. Responses obtained from the questionnaire will remain completely confidential and will be used only for scientific purposes. No fee will be requested from you during the study, and if you agree to respond, you will not be paid any fee.

- Indicates a required question

1. Please indicate your age. *
2. Please indicate your body weight (kg). *
3. Please indicate your height (cm). *
4. Are you regularly employed in a profession other than housewifery? (Single choice)

   ☐ Yes
   ☐ No
5. Please indicate your education level. (Single choice)

   ☐ Primary school
   ☐ Middle school
   ☐ High school
   ☐ Associate degree
   ☐ Bachelor's degree
   ☐ Graduate degree

6. Do you have any formal diagnoses related to shoulder, elbow, hand or wrist?

   ☐ Yes
   ☐ No

   If your answer to the above question is "Yes", please specify your diagnosis.

   ………….

7. Do you take any medication related to the diagnosis you mentioned above?

   ☐ Yes
   ☐ No

   If your answer to the above question is "Yes", please list the medication(s) you use.

.

8. How many years have you been married? *

……..

9. How many children do you have? *

………….

10. Which hand do you use more often in daily life? (Indicate your dominant hand.) *

- ☐ Right
- ☐ Left

11. Please indicate the intensity of your wrist pain. *

No pain at all Pain is unbearably severe

0 1 2 3 4 5 6 7 8 9 10

12. Please indicate the severity of fatigue you experience during the day. *

I do not feel tired at all. My fatigue is unbearably severe

0 1 2 3 4 5 6 7 8 9 10

PATIENT-BASED WRIST ASSESSMENT QUESTIONNAIRE

You will describe your average wrist complaints in the last week using a 0–10 scale. If you did not perform an activity in the last week, please state the level of expected strain or pain.

PAIN

At rest (while resting).

No pain Unbearable pain

0 1 2 3 4 5 6 7 8 9 10

When performing a job that involves repetitive wrist movements. *

No pain Unbearable pain

0 1 2 3 4 5 6 7 8 9 10

When lifting a heavy object. *

No pain Unbearable pain

0 1 2 3 4 5 6 7 8 9 10

How often do you experience your pain? *

Never Always

0 1 2 3 4 5 6 7 8 9 10

FUNCTION

A. Specific Activities

Turning a door handle using my affected hand. *

No difficulty at all Impossible to do

0 1 2 3 4 5 6 7 8 9 10

Cutting meat with a knife using my affected hand. *

No difficulty at all Impossible to do

0 1 2 3 4 5 6 7 8 9 10

Buttoning a shirt. *

No difficulty at all Impossible to do

0 1 2 3 4 5 6 7 8 9 10

Using the affected hand to push up from a chair (taking support from the affected hand). *

No difficulty at all Impossible to do

0 1 2 3 4 5 6 7 8 9 10

Carrying a 4.5 kilogram weight with the affected hand. *

No difficulty at all Impossible to do

0 1 2 3 4 5 6 7 8 9 10

Using toilet paper with the affected hand. *

No difficulty at all Impossible to do

0 1 2 3 4 5 6 7 8 9 10

B. Daily Activities

Personal care activities (dressing, showering). *

No difficulty at all Impossible to do

0 1 2 3 4 5 6 7 8 9 10

Household tasks (cleaning, maintenance). *

No difficulty at all Impossible to do

0 1 2 3 4 5 6 7 8 9 10

Work (your occupation or daily work). *

No difficulty at all Impossible to do

0 1 2 3 4 5 6 7 8 9 10

PAIN CATASTROPHIZING SCALE

Almost everyone has experienced pain at some point in life (headache, toothache, joint pain, muscle pain, etc.). We are interested in the emotions and thoughts you have when you experience pain. Below are 13 statements describing different emotions and thoughts related to pain. Using this scale, indicate the degree of the emotions and thoughts you have when you experience pain.

Leisure activities. *

- ☐ Not at all
- ☐ Mild degree
- ☐ Moderate degree
- ☐ Greatly
- ☐ Always

I worry constantly about whether the pain will ever stop. *

- ☐ Not at all
- ☐ Mild degree
- ☐ Moderate degree
- ☐ Greatly
- ☐ Always

I feel I cannot continue because of the pain. *

- ☐ Not at all
- ☐ Mild degree
- ☐ Moderate degree
- ☐ Greatly
- ☐ Always

I think the pain is terrible and will never get better. *

- ☐ Not at all
- ☐ Mild degree
- ☐ Moderate degree
- ☐ Greatly
- ☐ Always

The pain is a horrible thing and I feel overwhelmed by it. *

- ☐ Not at all
- ☐ Mild degree
- ☐ Moderate degree
- ☐ Greatly

☐ Always

I feel I cannot stand more pain. *

☐ Not at all
☐ Mild degree
☐ Moderate degree
☐ Greatly
☐ Always

I am afraid the pain will get worse. *

☐ Not at all
☐ Mild degree
☐ Moderate degree
☐ Greatly
☐ Always

I constantly think about other painful conditions. *

☐ Not at all
☐ Mild degree
☐ Moderate degree
☐ Greatly
☐ Always

I anxiously wish the pain to stop. *

☐ Not at all
☐ Mild degree
☐ Moderate degree
☐ Greatly
☐ Always

I cannot get the pain out of my mind. *

☐ Not at all
☐ Mild degree
☐ Moderate degree
☐ Greatly
☐ Always

I constantly think about how much the pain hurts me. *

☐ Not at all
☐ Mild degree
☐ Moderate degree
☐ Greatly
☐ Always

I keep thinking about how hard it is to wait for the pain to pass. *

- ☐ Not at all
- ☐ Mild degree
- ☐ Moderate degree
- ☐ Greatly
- ☐ Always

PAIN SELF-EFFICACY QUESTIONNAIRE

Rate how confident you are now that you can do the following despite your pain.

I can still enjoy things despite my pain. *

I have no confidence at all		I am completely confident

0 1 2 3 4 5 6

I can do most household tasks (tidying, washing up, etc.) despite my pain. *

I have no confidence at all		I am completely confident

0 1 2 3 4 5 6

I can socialize with friends or family as often as before despite my pain. *

I have no confidence at all		I am completely confident

0 1 2 3 4 5 6

In most situations I can cope with my pain. *

I have no confidence at all		I am completely confident

0 1 2 3 4 5 6

I can carry out a variety of tasks despite my pain (home or work tasks). *

I have no confidence at all		I am completely confident

0 1 2 3 4 5 6

I can still do many of the things I enjoy, such as hobbies and leisure activities, despite my pain. *

I have no confidence at all   I am completely confident

0 1 2 3 4 5 6

I can cope with my pain without medication. *

I have no confidence at all   I am completely confident

0 1 2 3 4 5 6

I can still achieve many of my goals despite my pain. *

I have no confidence at all   I am completely confident

0 1 2 3 4 5 6

I can maintain a normal lifestyle despite my pain. *

I have no confidence at all   I am completely confident

0 1 2 3 4 5 6

PATIENT HEALTH QUESTIONNAIRE-4 (PHQ-4)

Over the last 2 weeks, how often have you been bothered by the following problems? Please check the box that best represents your answer.

I have become gradually more active despite my pain. *

- ☐ Not at all
- ☐ Several days
- ☐ More than half the days
- ☐ Nearly every day

Feeling anxious, nervous, or on edge. *

- ☐ Not at all
- ☐ Several days
- ☐ More than half the days
- ☐ Nearly every day

Cannot stop or control worrying. *

- ☐ Not at all

- ☐ Several days
- ☐ More than half the days
- ☐ Nearly every day

Little interest or pleasure in doing things. *

- ☐ Not at all
- ☐ Several days
- ☐ More than half the days
- ☐ Nearly every day

Feeling down, depressed, or hopeless. *

- ☐ Not at all
- ☐ Several days
- ☐ More than half the days
- ☐ Nearly every day
